# Meta-analysis and time trend prediction of the prevalence of hypertension in Chinese college students

**DOI:** 10.1097/MD.0000000000035644

**Published:** 2023-10-20

**Authors:** Zhaoqi Zhang, Minghui Li, Jiatong Li, Ouyang Yu, Baoyuan Jiang, Yan Chen, Lianying Guo

**Affiliations:** a Department of Nutrition and Food Hygiene, School of Public Health, Shenyang Medical College, Shenyang, China; b Office, Panjin Center for Disease Control and Prevention, Panjin, Liaoning Province, China.

**Keywords:** college students, hypertension, meta-analysis, prevalence, trend analysis

## Abstract

**Background:**

To understand the prevalence of hypertension among Chinese college students over the past decade (2010–2020) and predict its future trend, we aim to provide a basis for preventing and controlling hypertension among college students.

**Methods:**

Databases such as Chinese National Knowledge Infrastructure, Wanfang database, PubMed, and Web of Science were searched, and publications on the prevalence of hypertension among Chinese college students from 2010 to 2020 were collected. Search for publications in both Chinese and English databases using keywords “hypertension,” “prevalence,” “disease status,” “cross-sectional survey,” “epidemiology,” “China,” “adolescents,” and “college students.” Publication screening, data extraction, and quality assessment were independently conducted by 2 researchers. Meta-analysis was performed using Stata 16, and trends in the prevalence of hypertension among college students were analyzed using R 4.2.0.

**Results:**

A total of 37 publications were included in this analysis, which involved 233,603 Chinese college students. The Meta-analysis results showed significant heterogeneity among the studies (I^2^ = 98.9%, *P < *.05). Using a random-effects model, the overall prevalence of hypertension among college students was estimated to be 3.3% (95% CI = 2.9%–3.6%), with a higher prevalence among male students (6.2%, 95% CI = 5.4%–7.1%) than female students (1.1%, 95% CI = 0.9%–1.3%). The prevalence of hypertension is notably higher in northern regions than in southern regions. The prevalence of hypertension among college students showed an increasing trend from 2010 to 2020. Trend analysis predicted that the prevalence of hypertension among college students will reach 10% and 14.6% by 2030 and 2040, respectively. The risk of hypertension in male students was 4.63 times higher than that of female students (95% CI = 2.97–7.23). Compared normal weight students, overweight and obese students had 3.08 times (95% CI = 2.48–3.82) and 6.69 times (95% CI = 2.25–19.90) higher risk of hypertension, respectively.

**Conclusion:**

The prevalence of hypertension in Chinese college students was about 3.3%. The prevalence of hypertension in male college students was higher than that in females, and the prevalence in northern regions was generally higher than that in southern regions. The prevalence of hypertension among Chinese college students will reach 10.0% in the next 10 years and 14.6% in the next 20 years. Male and BMI ≥ 24 were risk factors for hypertension among college students.

## 1. Introduction

Hypertension is a significant cardiovascular disease and a major global public health concern. Recent research suggested that the prevalence of hypertension was rapidly increasing among young adults in China.^[1]^ The trend was attributed to various factors such as prolonged sitting, reduced physical activity, heavy academic workload, fast-paced lifestyle, and unhealthy dietary habits. The gradual rise in hypertension prevalence among college students had posed a serious threat to their physical and mental health, necessitating the need for timely interventions and preventive measures.^[2]^

Despite numerous investigation reports on hypertension prevalence among college students, significant differences existed in reported prevalence rates due to variations in investigation regions, sample sizes, and time frames. For instance, Q Wen study^[3]^ at Tongling College in Anhui reported a hypertension detection rate of 16.4% among college students, while QQ Jiang cross-sectional study^[4]^ showed a 4.3% prevalence rate of hypertension among college students at a college in Wuhan. On the other hand, P. Hu physical examination survey^[5]^ at a medical college reported a meager 0.3% prevalence rate of hypertension among college students. Furthermore, there was limited research on temporal trends of hypertension prevalence among college students, which could hinder the development of public policies. To address this gap, this study aims to conduct a Meta-analysis to assess the prevalence rate, regional distribution differences, and temporal trends of hypertension among college students in China. Additionally, future trends will be predicted to provide a basis for the development of public policies for the prevention and management of hypertension among college students.

## 2. Materials and methods

### 2.1. Search strategy

Publications on the prevalence of hypertension among college students from 2010 to 2020 were retrieved from the Chinese National Knowledge Infrastructure, Wanfang Database, PubMed, and Web of Science databases. Search for publications in both Chinese and English databases using keywords “hypertension,” “prevalence,” “disease status,” “cross-sectional survey,” “epidemiology,” “China,” “adolescents,” and “college students.” The search strategy was jointly developed by researchers ZZ and GL and executed by ZZ across databases. Researchers ML and JT independently screened all the publications, following these specific steps: firstly, NoteExpress (Beijing Zhongwenzhuming Technology Co., Ltd) software was used to remove duplicate publications. Then, irrelevant publications were excluded based on titles and abstracts. Finally, the full text of the remaining publications was read, and relevant publications were re-selected based on inclusion and exclusion criteria. If there were disagreements during the publication selection, ZZ will read the full text of the disputed publication and strictly re-screened it according to the inclusion and exclusion criteria. Published publications were chosen; hence, ethical approval was not required.

### 2.2. Inclusion and exclusion criteria

This Meta-analysis included publications meeting the following inclusion criteria:

1. Complete and original data on hypertension prevalence among Chinese college students could be extracted from the publication;
2. The study subjects were college students;
3. The study was conducted in China;
4. If duplicate data was present, publication with a larger sample size was selected;
5. The publication research type was a cross-sectional survey;
6. Survey years were between 2010 to 2020.

The following exclusion criteria were applied:

1. The publication subjects did not include college students;
2. Relevant data could not be extracted;
3. The publication was conducted outside of China;
4. Hypertension was secondary to other diseases;
5. Duplicate research content was present in domestic or foreign publications.

### 2.3. High blood pressure diagnostic criteria

The definition of hypertension in this eta-analysis was in accordance with the Chinese Guidelines for the Prevention and Treatment of Hypertension (2018 revised edition),^[6]^ which states that hypertension was characterized by 3 non-same-day clinic measurements of systolic blood pressure ≥140 mm Hg and/or diastolic blood pressure ≥90 mm Hg in the absence of antihypertensive medication. For individuals with a history of hypertension taking antihypertensive medication, they should still be diagnosed with hypertension regardless of their measured blood pressure being lower than 140/90 mm Hg.

### 2.4. Data extraction

The final included publications were reviewed again by researchers Z.Z., L.G., and B.J., and data extraction was performed by researchers Z.Z. and B.J. During the data extraction process, the 2 researchers independently completed the data extraction for all the publications and cross-checked their results. In case of any discrepancies, researcher L.G. will re-read the publications with discrepancies and re-extracted the data from them. The extracted data included the first author, publication year, survey year, survey region, age, hypertension value, gender, total number of subjects, number of hypertension patients, blood pressure measurement, and prevalence rate. A summary table was filled out, and a database was established to organize the extracted data.

### 2.5. Quality evaluation

The quality of the publication included in the Meta-analysis was assessed using the Joanna Briggs Institute tool^[7]^ for evaluating the quality of prevalence studies. The evaluation form consisted of 9 items, each with 4 possible options: “Yes,” “No,” “Unclear,” and “Not Applicable.” A score of 1 was assigned for answering “Yes” and 0 for all other options. Publication with a score of ≥6 points was considered to be of high quality. Quality evaluation was conducted by researchers Y.O. and B.J.

### 2.6. Statistical analysis

In this manuscript, Stata 16.0 (StataCorp LLC) was utilized to conduct the Meta-analysis of hypertension prevalence among college students. If the heterogeneity test results showed I² < 50%, it was considered that either there was no heterogeneity or the heterogeneity among studies was small, and a fixed-effect model was applied. Conversely, a random-effects model was used if heterogeneity was present. Sensitivity analysis and publication bias tests were also performed on the publication. Additionally, trend analysis and prediction of hypertension prevalence rate among college students were conducted using R 4.2.0 software (R Foundation for Statistical Computing).

## 3. Results

### 3.1. Publication screening process and results

**Figure 1.**
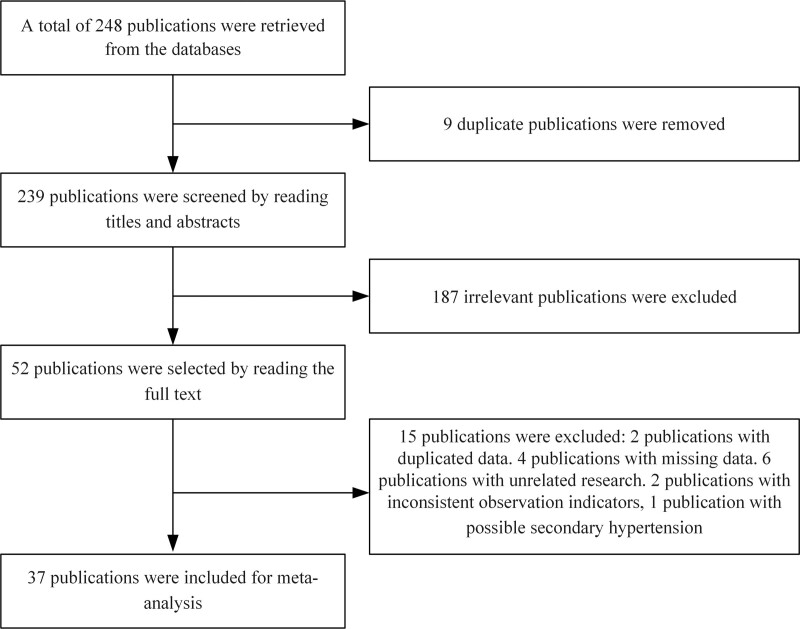
Meta-analysis publication search flowchart.

Initially, a total of 248 publication pertaining to hypertension prevalence among Chinese college students were identified, and after screening, 37 publications were included in the final analysis. The publication screening process and results are presented in Figure 1.

### 3.2. Basic characteristics and quality evaluation results of publication

All 37 publications included in this analysis were cross-sectional in nature and were conducted in 20 provinces or municipalities across China, comprising a total of 233,603 Chinese college students. Of these, 84,177 were male, 108,100 were female, and 41,326 were not differentiated by gender. In our manuscript, the age range of the Chinese college students we included was from 15 to 26 years old, with the majority of Chinese college students being concentrated around 18 years old. The researchers all used the same diagnostic criteria for hypertension, which defined it as systolic blood pressure ≥140 mm Hg and/or diastolic blood pressure ≥90 mm Hg. Most researchers chose to measure blood pressure using a sphygmomanometer on the right brachial artery or obtained blood pressure values from hospital physical examination reports, while a few researchers did not explicitly state this in their studies. For detailed information, please refer to Table 1. The quality of the publication included in this analysis was evaluated to be 6 points or higher. (see supplemental Table 1, http://links.lww.com/MD/K380)

**Table 1:** Basic characteristics and quality evaluation results of publication.

Study	Survey yr	Survey region	Age (yr)	SBP/DBP (mm Hg)	Blood pressure measurement methods	Blood pressure measurement positions	Gender	Sample size	Number of cases	Prevalence (%)	Quality score
W. Min (2012)^[26]^	2012	Guizhou	15–23	140/90	Mercury sphygmomanometer	Brachial artery on the right arm	Overall	4845	54	1.1	8
							Boy	1346	34	2.5	
							Girl	3499	20	0.6	
J.G. Ding (2013)^[27]^	2010–2011	Tianjin	18–24	140/90	Hospital physical examination records	Unclear	Overall	4834	85	1.8	8
W.L. Liu (2013)^[28]^	2010–2012	Tianjin	17–22	140/90	Mercury sphygmomanometer	Brachial artery on the right arm	Overall	9196	268	2.9	8
							Boy	3740	185	5	
							Girl	5456	83	1.5	
Y.M. Yang (2013)^[29]^	2011	Yunnan	15–27	140/90	Mercury sphygmomanometer	Brachial artery on the right arm	Overall	2400	12	0.5	9
							Boy	727	4	0.6	
							Girl	1673	8	0.5	
S.J. Li (2013)^[30]^	2012	Beijing	22–24	140/90	Electronic sphygmomanometer	Unclear	Overall	1649	59	3.6	9
							Boy	978	52	5.3	
							Girl	679	7	1	
Y. Gong (2013)^[31]^	2013	Gansu	18–25	140/90	Unclear	Unclear	Overall	3484	149	4.3	7
							Boy	1998	141	7.1	
							Girl	1486	8	0.5	
P. Liu (2014)^[32]^	2010–2012	Guangdong	17–23	140/90	Mercury sphygmomanometer	Brachial artery on the right arm	Overall	6214	318	5.1	7
Z.X. Fu (2014)^[33]^	2011	Anhui	17–23	140/90	Mercury sphygmomanometer	Unclear	Overall	5836	111	1.9	8
J.Z. Zhou (2014)^[17]^	2012	Hebei	18–22	140/90	Hospital physical examination records	Brachial artery on the right arm	Overall	3560	152	4.3	9
							Boy	1955	105	5.4	
							Girl	1605	47	2.9	
H.B. Lin (2014)^[34]^	2013	Jilin	16–24	140/90	Electronic sphygmomanometer	Brachial artery	Overall	3017	62	2	8
							Boy	1923	46	2.4	
							Girl	1148	16	1.4	
F.N. Cui (2016)^[35]^	2012	Beijing	14–26	140/90	Mercury sphygmomanometer	Brachial artery on the right arm	Overall	2744	47	1.7	9
							Boy	1046	42	4	
							Girl	1698	5	0.3	
Y.Y. Wang (2016)^[15]^	2014	Henan	19.0 ± 1.21	140/90	Electronic sphygmomanometer	Brachial artery on the right arm	Overall	1057	17	1.6	8
							Boy	94	4	4.3	
							Girl	963	13	1.4	
J. Ding (2016)^[36]^	2015	Jiangsu	College student	140/90	Unclear	Brachial artery on the right arm	Overall	4660	136	2.9	9
							Boy	2910	132	4.5	
							Girl	1790	4	0.2	
L. Zhao (2016)^[37]^	2015	Jilin	College student	140/90	Electronic sphygmomanometer	Brachial artery on the right arm	Overall	695	38	5.5	9
							Boy	235	37	15.7	
							Girl	460	1	0.2	
P. Pan (2016)^[38]^	2016	Henan	16–22	140/90	Hospital physical examination records	Unclear	Overall	12601	118	0.9	9
							Boy	7632	11	0.2	
							Girl	4969	107	1.4	
P. Hu (2017)^[5]^	2010–2015	Anhui	17–20	140/90	Hospital physical examination records	Unclear	Overall	19883	49	0.3	6
							Boy	7827	33	0.4	
							Girl	12056	16	0.1	
G.Y. Chen (2017)^[12]^	2013–2015	Guangdong	19.5 ± 1.26	140/90	Hospital physical examination records	Unclear	Overall	45160	201	0.5	8
							Boy	17342	162	0.9	
							Girl	27818	39	0.1	
F. Yang (2017)^[11]^	2014	Guizhou	16–25	140/90	Mercury sphygmomanometer	Brachial artery on the right arm	Overall	3675	12	0.3	9
							Boy	1545	10	0.6	
							Girl	2130	2	0.1	
J.P. Liu (2017)^[10]^	2015	Hunan	17–26	140/90	Mercury sphygmomanometer	Brachial artery on the right arm	Overall	1719	182	10.6	8
F.Y. Yan (2017)^[16]^	2017	Gansu	College student	140/90	Mercury sphygmomanometer	Brachial artery on the right arm	Overall	1320	67	5.1	9
							Boy	700	58	8.3	
							Girl	620	9	1.5	
L.F. Yang (2018)^[9]^	2016	Anhui	15–26	140/90	Mercury sphygmomanometer	Unclear	Overall	4873	667	13.7	9
							Boy	2923	600	20.5	
							Girl	1950	67	3.4	
J.M. Lu (2018)^[39]^	2016–2017	Shaanxi	17–23	140/90	Hospital physical examination records	Unclear	Overall	6252	63	1	9
							Boy	2093	37	1.8	
							Girl	4159	26	0.6	
Q. Meng (2018)^[40]^	2018	Jiangsu	19–23	140/90	Unclear	Unclear	Overall	500	16	3.2	7
X.L. Li (2019)^[41]^	2017	Hebei	19 ± 1.49	140/90	Mercury sphygmomanometer	Brachial artery on the right arm	Overall	952	59	6.2	8
J. Wang (2019)^[42]^	2018	Chongqing	16–23	140/90	Hospital physical examination records	Unclear	Overall	4576	28	0.6	9
							Boy	2897	23	0.8	
							Girl	1679	5	0.3	
M. Zhang (2019)^[43]^	2018	Anhui	College student	140/90	Hospital physical examination records	Unclear	Overall	8569	276	3.2	7
K. Duan (2019)^[44]^	2018	Hubei	College student	140/90	Physical examination by trained investigator	Unclear	Overall	2150	223	10.4	7
							Boy	1180	188	15.9	
							Girl	970	35	3.6	
X.Y. Ji (2020)^[8]^	2018	Henan	18–27	140/90	Electronic sphygmomanometer	Brachial artery on the right arm	Overall	5185	723	13.9	9
							Boy	2051	593	28.9	
							Girl	3134	130	4.1	
X.P. Song (2020)^[45]^	2019	Inner Mongolia	College student	140/90	Electronic sphygmomanometer	Brachial artery on the right arm	Overall	4830	285	5.9	9
							Boy	1911	217	11.4	
							Girl	2919	68	2.3	
Q.X. Deng (2020)^[14]^	2020	Shandong	18.2 ± 0.79	140/90	Hospital physical examination records	Unclear	Overall	5465	139	2.5	8
							Boy	2385	103	4.3	
							Girl	3080	36	1.2	
Q.Q. Jiang (2021)^[4]^	2015–2017	Hubei	College student	140/90	Electronic sphygmomanometer	Brachial artery	Overall	12849	558	4.3	9
							Boy	5652	445	7.9	
							Girl	7197	113	1.6	
X.D. Guan (2021)^[46]^	2015–2019	Liaoning	18.8 ± 1.13	140/90	Hospital physical examination records	Unclear	Overall	8496	108	1.3	8
							Boy	1923	88	4.6	
							Girl	6573	20	0.3	
G.S. Hou (2021)^[47]^	2016–2020	Beijing	21–22	140/90	Electronic sphygmomanometer	Brachial artery on the right arm	Overall	14350	717	5	9
							Boy	7270	627	8.6	
							Girl	7080	90	1.3	
W. Yang (2021)^[48]^	2017	Tibet	18–21	140/90	Unclear	Brachial artery on the right arm	Overall	2533	37	1.5	8
J. Huang (2021)^[49]^	2018–2020	Jiangsu	18–20	140/90	Hospital physical examination records	Unclear	Overall	10003	509	5.1	8
Q. Wen (2021)^[3]^	2019	Anhui	17–24	140/90	Hospital physical examination records	Unclear	Overall	268	44	16.4	6
N. Zhang (2021)^[50]^	2019	Zhejiang	18.2 ± 0.54	140/90	Mercury sphygmomanometer	Brachial artery on the right arm	Overall	3203	49	1.5	9
							Boy	1894	39	1.2	
							Girl	1309	10	0.3	

DBP = diastolic blood pressure, SBP = systolic blood pressure.

### 3.3. Meta-analysis of the prevalence of hypertension

#### 3.3.1. The overall prevalence of hypertension.

**Figure 2.**
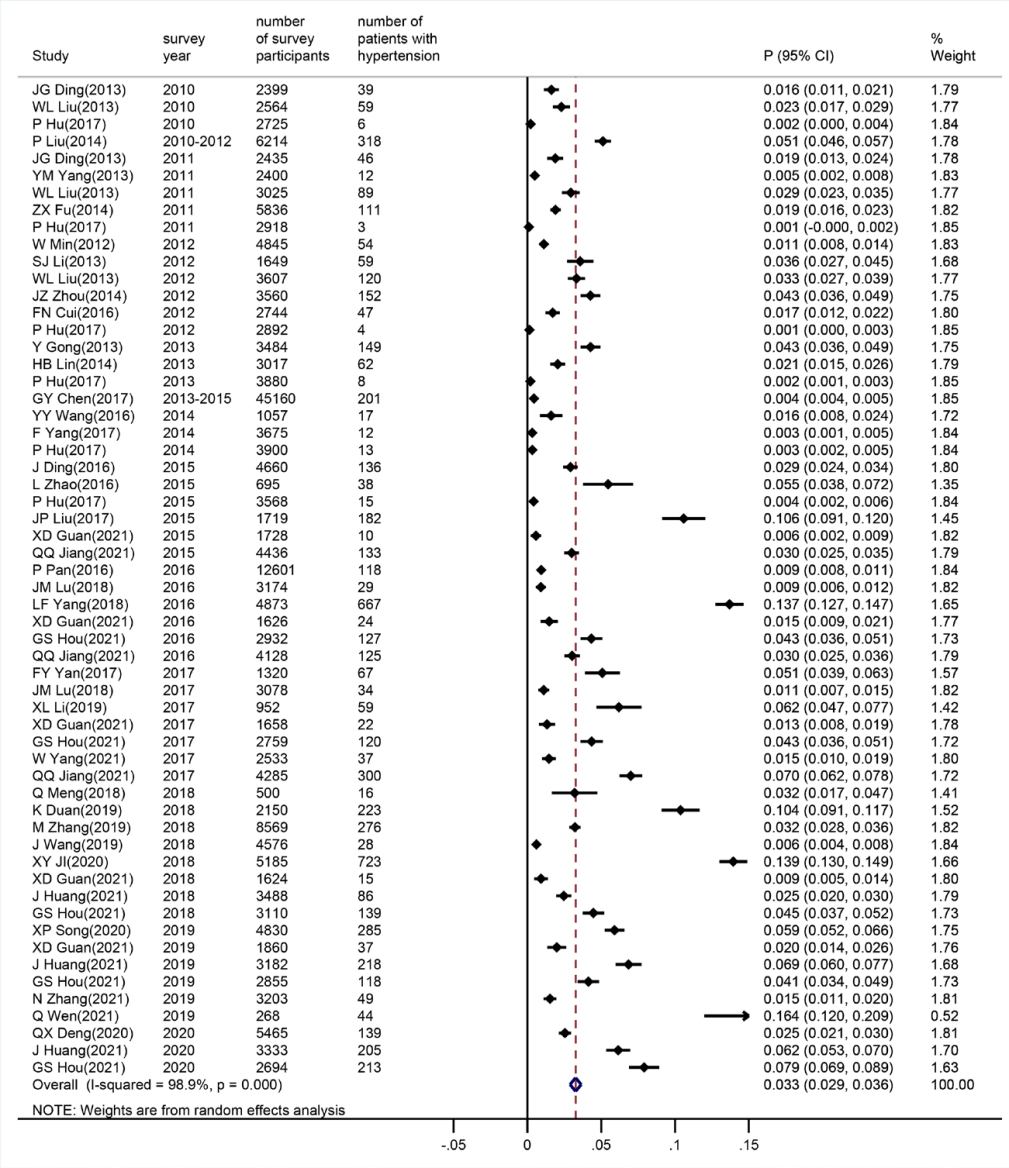
Meta-analysis forest plot of hypertension prevalence among Chinese college students.

Among the 37 included studies, the prevalence of hypertension among Chinese college students ranged from 0.3% to 16.4%. Meta-analysis showed significant heterogeneity between studies (I² = 98.9%, *P* < .05), therefore a random-effects model was used. The overall prevalence of hypertension among college students was 3.3% (95% CI = 2.9%–3.6%), as shown in Figure 2. Sensitivity analysis indicated good stability of the 37 studies, although the funnel plot showed some publication bias, we also conducted a subgroup analysis and created a corresponding forest plot. (see supplemental Figure 1, http://links.lww.com/MD/K366, 2, http://links.lww.com/MD/K367, 3, http://links.lww.com/MD/K368)

#### 3.3.2. The prevalence of hypertension among college students of different genders.

The analysis of hypertension prevalence among college students by gender revealed that male college students had a prevalence rate of 6.2% (95% CI = 5.4%–7.1%), while female college students had a prevalence rate of 1.1% (95% CI = 0.9%–1.3%), indicating a higher prevalence rate of hypertension among male college students compared to their female counterparts. (see supplemental Figure 4, http://links.lww.com/MD/K369)

#### 3.3.3. The prevalence of hypertension among college students in different survey regions.

Upon analyzing hypertension prevalence among college students across 20 provinces or municipalities, it was observed that southern regions exhibited relatively low prevalence. Notably, Chongqing and Yunnan recorded prevalence of 0.6% and 0.5%, respectively. Conversely, northern regions presented relatively high prevalence. Inner Mongolia, Hebei, and Beijing recorded prevalence of 5.9%, 5.1%, and 4.3%, respectively, excluding Hunan, where the prevalence soared to 10.6% (see supplemental Figure 5, http://links.lww.com/MD/K370)

#### 3.3.4. The prevalence of hypertension among college students in different survey years.

**Figure 3.**
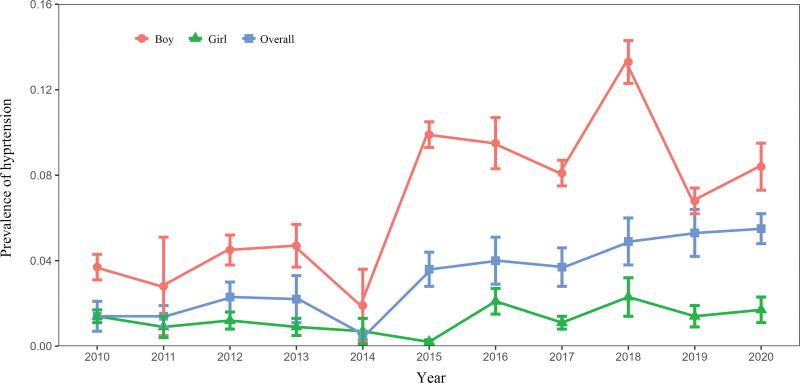
Analysis of hypertension prevalence among college students in different survey years.

The analysis of the prevalence of hypertension among college students across different survey years revealed a declining trend in overall prevalence from 2012 to 2014 and from 2016 to 2017. However, excluding these years, the prevalence displayed an upward trend, with the highest prevalence recorded in 2020, reaching 5.5%. The highest prevalence of hypertension among male and female students both occurred in 2018, with male students at 13.3% and female students at 2.3%. The lowest prevalence among male students occurred in 2014, at 1.9%, while the lowest prevalence among female students occurred in 2015, at 0.2%, as shown in Figure 3. Notably, the prevalence of hypertension among male college students was higher than the overall prevalence, while the prevalence of hypertension among female college students was lower than the overall prevalence. (see supplemental Figure 6, http://links.lww.com/MD/K371, 7, http://links.lww.com/MD/K372, 8, http://links.lww.com/MD/K373)

### 3.4. Trend analysis of hypertension prevalence

#### 3.4.1. Model selection and diagnosis.

To predict the trend of hypertension prevalence among Chinese college students in the forthcoming 2 decades, the statistical software R 4.2.2 was employed, and a scatter plot was generated, revealing a linear trend. Subsequently, normality tests were conducted on the independent variable (year) and the dependent variable (hypertension prevalence), and both were found to exhibit a normal distribution. The correlation between the 2 variables was examined using the Pearson correlation test, indicating a statistically significant correlation (*P* < .05). The predictive significance of the model was analyzed, and the results indicated *R* = 0.6685, *P* < .05, signifying the model predictive capability. The residual and fitted value plot demonstrated a symmetric residual distribution centered around zero, signifying a good model fit. The Q-Q plot followed a normal distribution, while the standardized residual and fitted value plot showed that the residuals satisfied the homoscedasticity assumption, except for 3 poorly fitted values. Furthermore, the residual and leverage plot were utilized to detect outliers, high leverage points, and strong influence points, which were subsequently confirmed using R. Results revealed that there were no outliers or high leverage points; however, the first, fifth, and eleventh values were identified as strong influence points. In conclusion, the prediction model was deemed feasible.

#### 3.4.2. Trend analysis.

**Figure 4.**
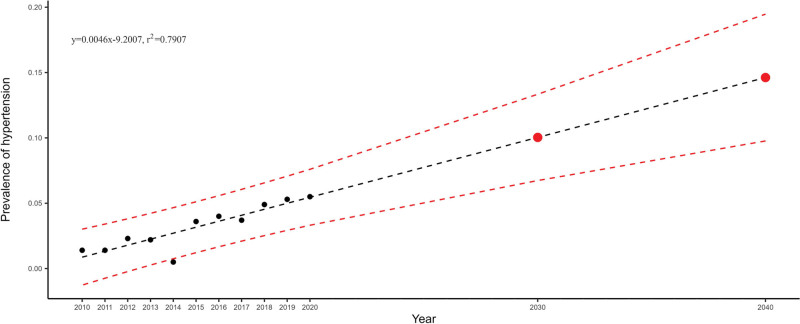
Trend analysis of hypertension prevalence among college students.

The employment of linear regression enabled the derivation of the slope and intercept for hypertension prevalence among college students, resulting in the equation y = 0.0039 × −7.827. This equation implied that hypertension prevalence among college students is on the rise, as illustrated in Figure 4. Employing this equation for predictive purposes indicated that by 2030 and 2040, the prevalence of hypertension among college students was estimated to be approximately 10% and 14.6%, respectively.

### 3.5. The analysis of hypertension associated factors

**Figure 5.**
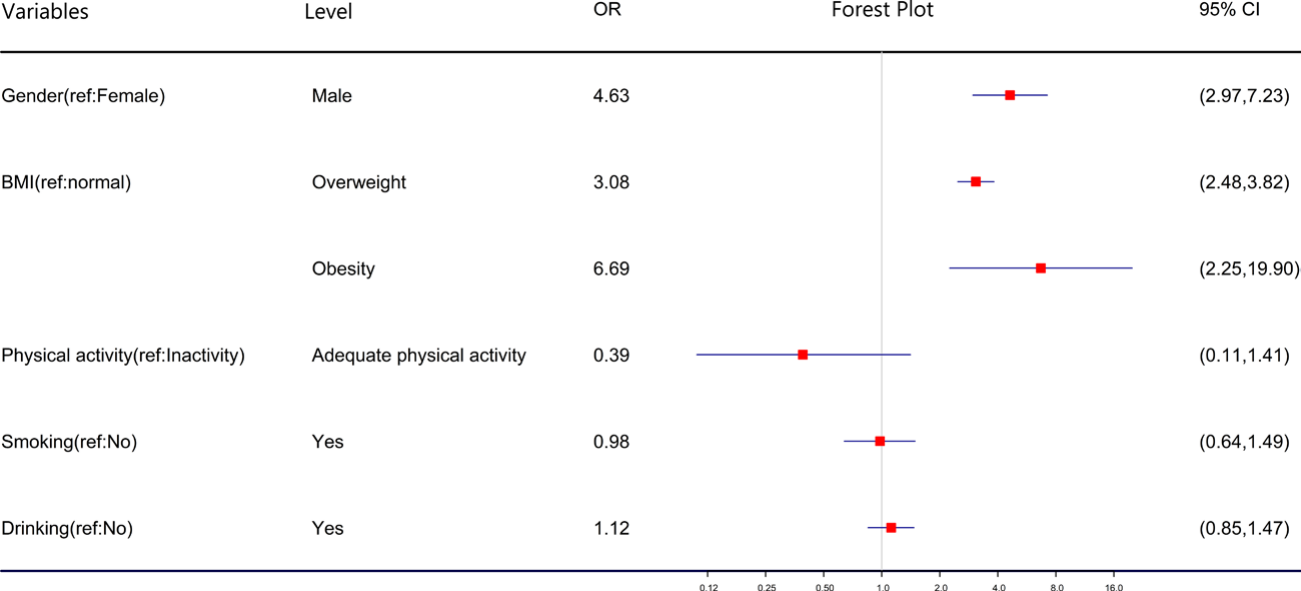
The Meta-analysis forest plot of hypertension associated factors. * BMI of 18.5 ≤ BMI < 24 is considered normal, 24 ≤ BMI < 28 is considered overweight, and BMI ≥ 28 is considered obesity. Adequate physical activity is defined as engaging in physical activity at least 3 times per week. Smoking is defined as smoking at least 1 cigarette per day for a duration of 6 mo or more. Drinking is defined as consuming alcohol more than once a week for a duration of 1 yr or more. BMI = body mass index.

Through a meta-analysis of hypertension associated factors, we found that among college students, male students had a higher risk of hypertension compared to female students, with a risk that was 4.63 times higher (95% CI = 2.97–7.23). Additionally, being overweight and obese would increase the risk of hypertension in college students. Specifically, overweight students had a risk that was 3.08 times higher (95% CI = 2.48–3.82) than normal students, while obese students had a risk that was 6.69 times higher (95% CI = 2.25–19.90) than normal students, as illustrated in Figure 5 However, in this manuscript, smoking, alcohol consumption, and lack of exercise were not identified as risk factors for hypertension among college students. (see supplemental Figure 9, http://links.lww.com/MD/K374, 10, http://links.lww.com/MD/K375, 11, http://links.lww.com/MD/K376, 12, http://links.lww.com/MD/K377, 13, http://links.lww.com/MD/K378, 14, http://links.lww.com/MD/K379)

## 4. Discussion

Hypertension, a global health issue of paramount significance, posed serious health risks to people. Alarmingly, there was mounting evidence to suggest a rising prevalence of hypertension among Chinese college students, urging the need for timely interventions. Early identification and management of this public health problem are imperative to curb its detrimental consequences. Our study extracted the prevalence data of hypertension among college students from 2010 to 2020, as there were numerous research papers related to hypertension in China, but there had been scarce meta-analyses on hypertension in recent years, especially in relation to the college student population. The publications included in this study reached a consensus on the diagnostic threshold for hypertension, with accurate measurement methods, proper instruments, and trained operators, resulting in high reliability. This study provided a meticulous analysis of hypertension prevalence among Chinese college students, uncovering a worrisome prevalence rate of 3.3%. However, the findings from other studies highlighted a much higher prevalence of hypertension among college students. X.Y. Ji study,^[8]^ for instance, uncovered an alarming hypertension prevalence rate of 13.9% among college students in 3 colleges located in Zhengzhou in 2018. Similarly, L.F. Yang investigation^[9]^ found that the prevalence of hypertension among college students in a college in Fuyang in 2017 was as high as 13.7%. Additionally, J.P. Liu study^[10]^ conducted in 2015 reported a prevalence rate of 10.6% among college students in a college in Xiangnan, while Q Wen study^[3]^ of Tongling College revealed an alarming prevalence rate of 16.4%. The reasons for the differences in the prevalence rates of different studies might be that college students in X.Y. Ji study generally had unhealthy diets and lack of sleep, there was a higher proportion of male students in L.F. Yang survey, Xiangnan region surveyed by J.P. Liu belongs to the central region with a high-salt diet habit, and most students in Q Wen study were overweight and obese. Some reports suggested that the prevalence of hypertension was low among college students. For instance, P. Hu conducted a study^[5]^ on the physical examination results of a medical college from 2010 to 2015, and the prevalence of hypertension was found to be about 0.3%. In 2017, F. Yang conducted a hypertension study^[11]^ on college students in Guizhou, and the prevalence of hypertension was also found to be 0.3%. G.Y. Chen and colleagues conducted a survey^[12]^ on the prevalence of hypertension among college students in the western Guangdong province from 2013 to 2015, and the prevalence was about 0.5%. The low prevalence might be attributed to the fact that P. Hu and F. Yang surveyed freshmen who experience relatively less academic pressure, while G.Y. Chen surveyed the western Guangdong province where the diet was light and low in salt and oil. These results indicated that the prevalence of hypertension among college students might be influenced by many external factors. Previous research had elucidated that obesity, excessive salt intake, high levels of mental stress, inadequate sleep quality, and a lack of physical activity were all contributing factors to the development of hypertension in adolescents.^[13]^ The findings presented indicated that adopting a favorable lifestyle might serve as a crucial factor in mitigating the onset of hypertension among individuals enrolled in college. Consequently, incorporating health education programs tailored to address this issue might serve as an effective preventive measure. The implementation of such schooling at an early stage might prove to be more efficacious in mitigating the prevalence rate of hypertension.

The sub-analysis findings revealed that male college students exhibited a higher prevalence of hypertension in comparison to their female counterparts, with rates of 6.2% and 1.1%, respectively. This corresponds with prior research conducted by esteemed scholars such as Q.X. Deng,^[14]^ Y.Y. Wang,^[15]^ F.Y. Yang,^[16]^ J.Z. Zhou,^[17]^ and Q.Q. Jiang,^[4]^ among others. Furthermore, our meta-analysis results on the associated factors of hypertension indicated that male was a risk factor for hypertension, which was consistent with the observed gender differences in disease prevalence. Research suggested that estrogen in women conferred the capacity to regulate vascular activity and safeguard blood vessels, thereby diminishing vascular tension, enhancing endothelial cell function, and forestalling hypertension.^[18,19]^ Meanwhile the dissimilar dietary patterns and lifestyle habits observed in male and female students might provide a plausible explanation for the diverging prevalence rates. Males tended to gravitate towards a diet that was rich in high-calorie, high-fat, and high-salt foods, whereas females tended to prioritize a more wholesome and nutritionally balanced diet. Research suggested that obesity and overweight were more frequently detected among male students in certain colleges, which, in turn, was linked with the occurrence of hypertension as obesity constitutes a primary risk factor for hypertension.^[20,21]^ Regarding overweight and obesity, our study also addressed them in the meta-analysis of hypertension associated factors, and the results indicated that having a BMI above 24 would become an important risk factor for hypertension. Compared to females, males tended to develop unhealthy habits such as staying up late, smoking, drinking alcohol, and excessive use of electronic devices.^[22,23]^ However, the meta-analysis results of our study on the associated factors of hypertension did not include smoking and drinking as risk factors. This might be due to the fact that the sample of college students in this study was primarily focused on 18-year-olds, who were relatively young and had not yet developed or been exposed to unhealthy habits, or it could be due to a smaller sample size in the factors being studied. Therefore, the above results suggested that we could focus on gender and BMI for targeted prevention and treatment of hypertension in males, overweight, or obese individuals.

The prevalence of hypertension among college students exhibited significant regional disparities. Specifically, the overall prevalence rate of hypertension was higher in northern regions, such as Beijing, Inner Mongolia, Gansu, Shandong, and Henan, than in other southern regions. Moreover, Hunan mirrors the findings of QQ Jiang investigation,^[4]^ indicating that students residing in central regions encounter a greater risk of hypertension, potentially due to prolonged exposure to spicy and high-salt diets. Research suggested that differences in geography, dietary habits, dietary structure, and lifestyles between northern and southern regions could account for the observed outcomes.^[24]^ Additionally, distinct levels of economic development and awareness of hypertension across regions could potentially influence the prevalence of hypertension.^[25]^

The manuscript analyzed the trend of hypertension prevalence among college students from 2010 to 2020. The findings revealed an overall increasing trend in hypertension prevalence, corroborating the results of prior studies conducted by esteemed scholars such as X.D. Guan, J.M. Lu, G.S. Hou, W.L. Liu, and Q.Q. Jiang. Furthermore, the analysis of hypertension prevalence projections over the next 2 decades indicated a continued upward trajectory, with anticipated rates of 10% and 14.6% in 2030 and 2040, respectively. These results underscored the importance of implementing health education initiatives aimed at hypertension prevention among college students as early as possible to mitigate the growing burden of hypertension in this population.

The present study served to bridge the gap in the existing publication on hypertension prevalence among college students and predictions were made for the future prevalence of hypertension among college students, providing scientific evidence for the development of public health policies for the prevention and control of hypertension in college students. A large-scale sample comprising 233,603 Chinese college students was incorporated into the study, ensuring the robustness and reliability of the findings. Notwithstanding, several limitations had to be acknowledged. Firstly, the high heterogeneity among the included studies and potential publication bias might have compromised the stability of the results. Secondly, the low inclusion of publication or sample size in some studies examining hypertension prevalence across different regions and years could weaken the reliability of the results and potentially introduce biases. Meanwhile, insufficient sample size during the meta-analysis of associated factors might lower the credibility of the results.

## 5. Conclusions

Based on the present study findings, the estimated prevalence of hypertension among Chinese college students was approximately 3.3%, with male students exhibiting a higher prevalence rate than their female counterparts. Additionally, the prevalence of hypertension is notably higher in northern regions than in southern regions. Alarmingly, predictions suggest that the prevalence of hypertension among Chinese college students will surge to 10.0% in the next decade and 14.6% in the following 2 decades, highlighting the gravity of the situation. Male and BMI ≥ 24 were risk factors for hypertension among college students. Consequently, relevant authorities must prioritize the implementation of health education initiatives targeting college students to curb or prevent hypertension prevalence effectively.

## Author contributions

**Conceptualization:** Zhaoqi Zhang, Yan Chen, Lianying Guo.

**Data curation:** Zhaoqi Zhang, Minghui Li, Jiatong Li, Ouyang Yu, Baoyuan Jiang, Lianying Guo.

**Formal analysis:** Minghui Li, Baoyuan Jiang.

**Resources:** Jiatong Li.

**Software:** Zhaoqi Zhang, Minghui Li, Jiatong Li, Lianying Guo.

**Supervision:** Zhaoqi Zhang, Baoyuan Jiang, Yan Chen, Lianying Guo.

**Validation:** Ouyang Yu.

**Visualization:** Baoyuan Jiang.

**Writing – original draft:** Zhaoqi Zhang.

**Writing – review & editing:** Yan Chen, Lianying Guo.

## Supplementary Material






























