# Supplementary material for: Meta-analysis and time trend prediction of the prevalence of hypertension in Chinese college students
Source: Medicine (Baltimore). 2023 Oct 20;102(42):e35644. doi: 10.1097/MD.0000000000035644 (PMC10589676; doi:10.1097/MD.0000000000035644)
Supplement: Supplementary file 1 [file medi-102-e35644-s001.docx]

| Table1 Quality assessment of included studies using the Joanna Briggs Institute tool. | | | | | | | | | | |
| --- | --- | --- | --- | --- | --- | --- | --- | --- | --- | --- |
| study | Is the sampling frame suitable for the target population? | Was the appropriate method used to sample the study subjects? | Is the sample size sufficient? | Have the study subjects and research location been described in detail? | Is there sufficient coverage of the study subjects for data analysis? | Have effective methods been used to identify disease or health problems? | Have standard and reliable methods been used to measure the study subjects? | Is the data analysis method appropriate? | Is the response rate sufficient, and if not, have appropriate methods been used to address it? | score |
| W Min,2012 | No | Yes | Yes | Yes | Yes | Yes | Yes | Yes | Yes | 8 |
| JG Ding,2013 | Yes | Yes | Yes | Yes | No | Yes | Yes | Yes | Yes | 8 |
| WL Liu,2013 | Yes | Yes | Yes | Yes | No | Yes | Yes | Yes | Yes | 8 |
| YM Yang,2013 | Yes | Yes | Yes | Yes | Yes | Yes | Yes | Yes | Yes | 9 |
| SJ Li,2013 | Yes | Yes | Yes | Yes | Yes | Yes | Yes | Yes | Yes | 9 |
| Y Gong,2013 | Yes | Yes | Yes | No | Yes | Yes | Yes | Unclear | Yes | 7 |
| P Liu,2014 | Yes | No | Yes | No | Yes | Yes | Yes | Yes | Yes | 7 |
| ZX Fu,2014 | Yes | Yes | Yes | Yes | Yes | Yes | Yes | Yes | No | 8 |
| JZ Zhou,2014 | Yes | Yes | Yes | Yes | Yes | Yes | Yes | Yes | Yes | 9 |
| HB Lin,2014 | Yes | Yes | Yes | Yes | Yes | Yes | Unclear | Yes | Yes | 8 |
| FN Cui,2016 | Yes | Yes | Yes | Yes | Yes | Yes | Yes | Yes | Yes | 9 |
| YY Wang,2016 | Yes | Yes | Yes | Yes | No | Yes | Yes | Yes | Yes | 8 |
| J Ding,2016 | Yes | Yes | Yes | Yes | Yes | Yes | Yes | Yes | Yes | 9 |
| L Zhao,2016 | Yes | Yes | Yes | Yes | Yes | Yes | Yes | Yes | Yes | 9 |
| P Pan,2016 | Yes | Yes | Yes | Yes | Yes | Yes | Yes | Yes | Yes | 9 |
| P Hu,2017 | Yes | Yes | Yes | No | Yes | No | Yes | No | Yes | 6 |
| GY Chen,2017 | Yes | No | Yes | Yes | Yes | Yes | Yes | Yes | Yes | 8 |
| F Yang,2017 | Yes | Yes | Yes | Yes | Yes | Yes | Yes | Yes | Yes | 9 |
| JP Liu,2017 | Yes | Yes | Yes | No | Yes | Yes | Yes | Yes | Yes | 8 |
| FY Yan,2017 | Yes | Yes | Yes | Yes | Yes | Yes | Yes | Yes | Yes | 9 |
| LF Yang,2018 | Yes | Yes | Yes | Yes | Yes | Yes | Yes | Yes | Yes | 9 |
| JM Lu,2018 | Yes | Yes | Yes | Yes | Yes | Yes | Yes | Yes | Yes | 9 |
| Q Meng,2018 | Yes | Yes | No | Yes | Yes | Yes | Yes | No | Yes | 7 |
| XL Li,2019 | Yes | Yes | No | Yes | Yes | Yes | Yes | Yes | Yes | 8 |
| J Wang,2019 | Yes | Yes | Yes | Yes | Yes | Yes | Yes | Yes | Yes | 9 |
| M Zhang,2019 | Yes | Yes | Yes | No | No | Yes | Yes | Yes | Yes | 7 |
| K Duan,2019 | Yes | Yes | Yes | No | Yes | Yes | No | Yes | Yes | 7 |
| XY JI,2020 | Yes | Yes | Yes | Yes | Yes | Yes | Yes | Yes | Yes | 9 |
| XP Song,2020 | Yes | Yes | Yes | Yes | Yes | Yes | Yes | Yes | Yes | 9 |
| QX Deng,2020 | Yes | Yes | Yes | Yes | Yes | Yes | Yes | Yes | No | 8 |
| QQ Jiang,2021 | Yes | Yes | Yes | Yes | Yes | Yes | Yes | Yes | Yes | 9 |
| XD Guan,2021 | Yes | Yes | Yes | No | Yes | Yes | Yes | Yes | Yes | 8 |
| GS Hou,2021 | Yes | Yes | Yes | Yes | Yes | Yes | Yes | Yes | Yes | 9 |
| W Yang,2021 | Yes | Yes | Yes | Yes | No | Yes | Yes | Yes | Yes | 8 |
| J Huang,2021 | Yes | No | Yes | Yes | Yes | Yes | Yes | Yes | Yes | 8 |
| Q Wen,2021 | Yes | Yes | No | Yes | No | Yes | Yes | No | Yes | 6 |
| N Zhang,2021 | Yes | Yes | Yes | Yes | Yes | Yes | Yes | Yes | Yes | 9 |
