# Supplementary figures and images for: Meta-analysis and time trend prediction of the prevalence of hypertension in Chinese college students
Source: Medicine (Baltimore). 2023 Oct 20;102(42):e35644. doi: 10.1097/MD.0000000000035644 (PMC10589676; doi:10.1097/MD.0000000000035644)

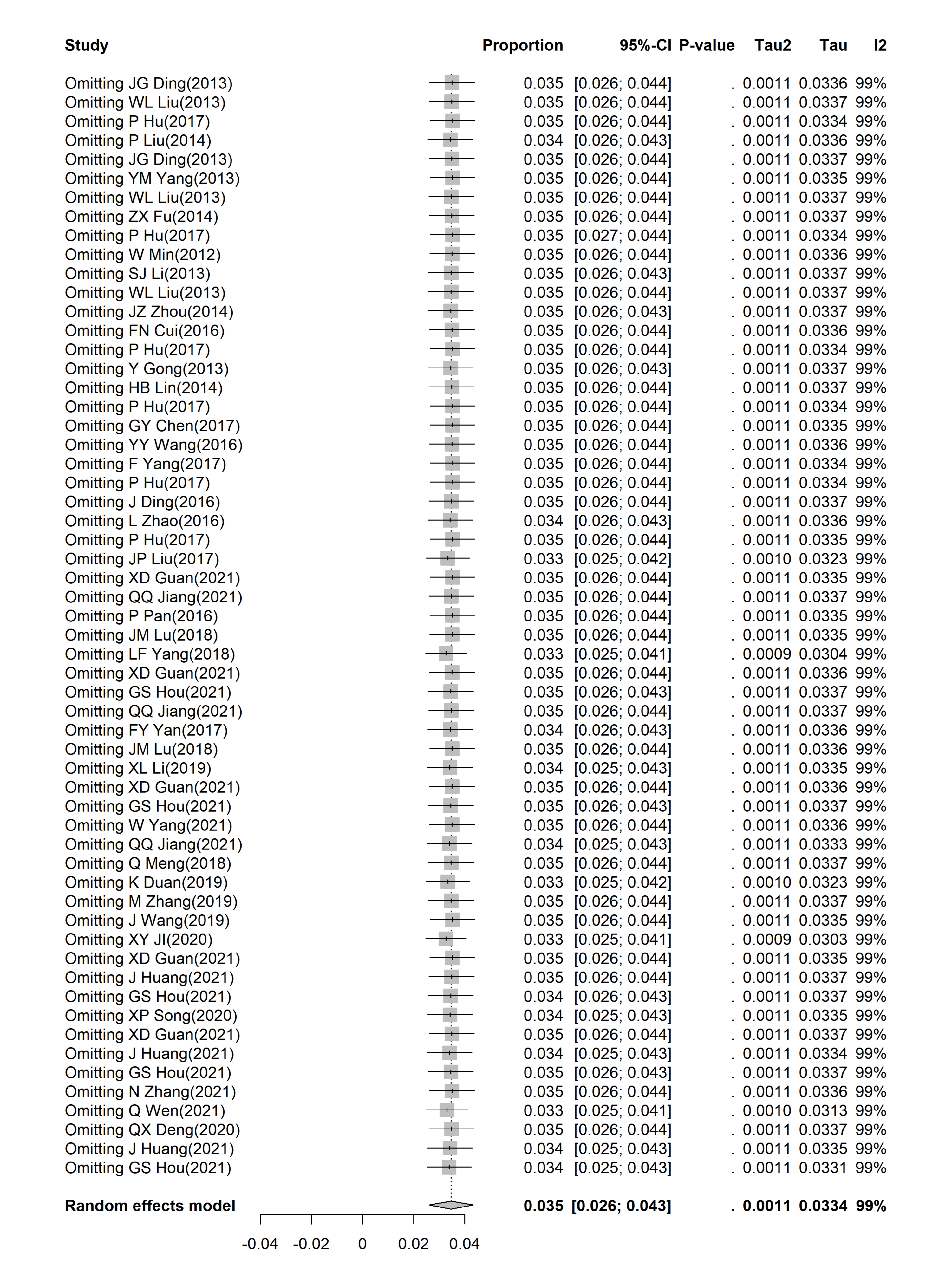

Supplement: Supplementary file 2 [file medi-102-e35644-s002.tif]

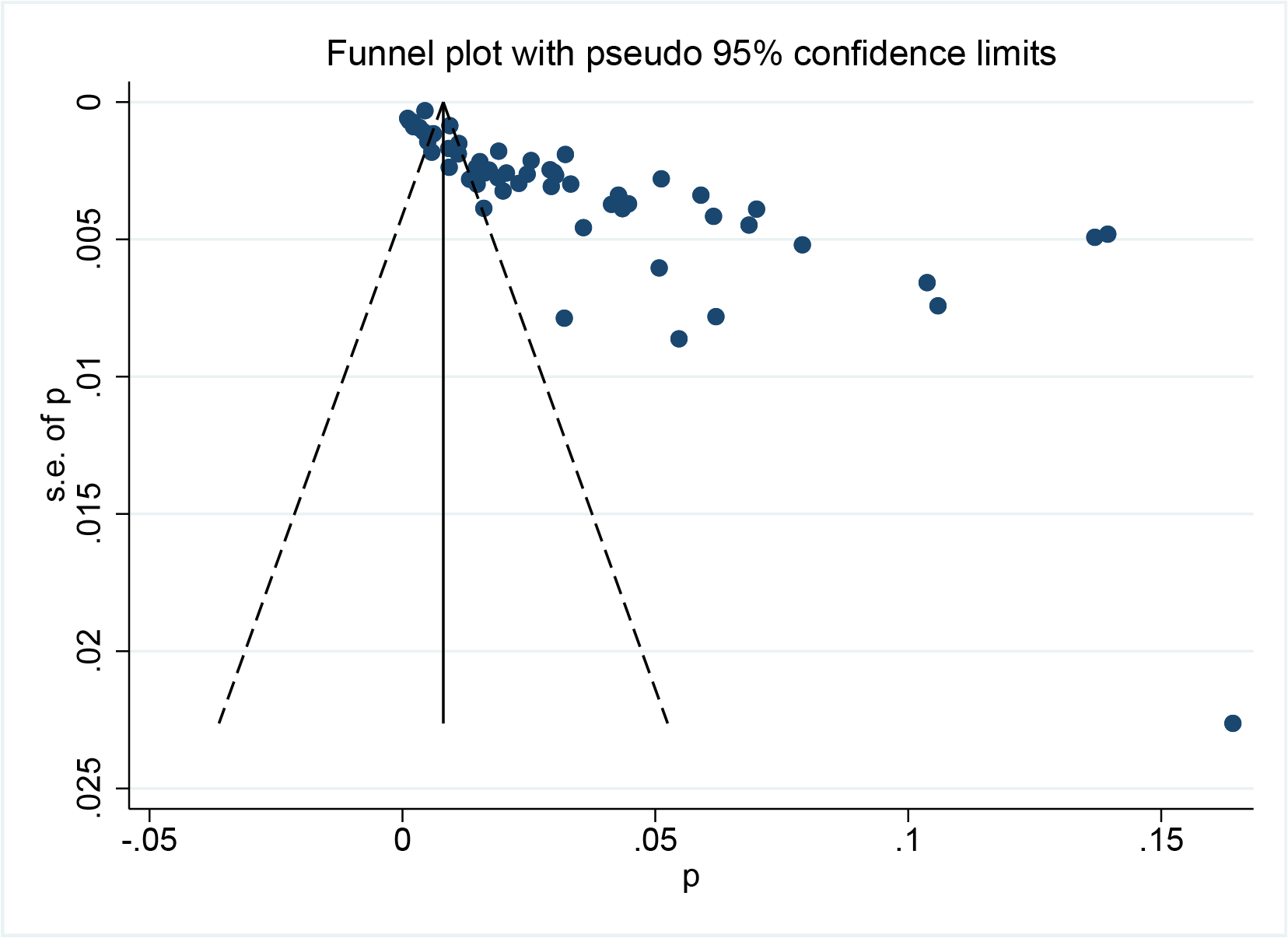

Supplement: Supplementary file 3 [file medi-102-e35644-s003.tif]

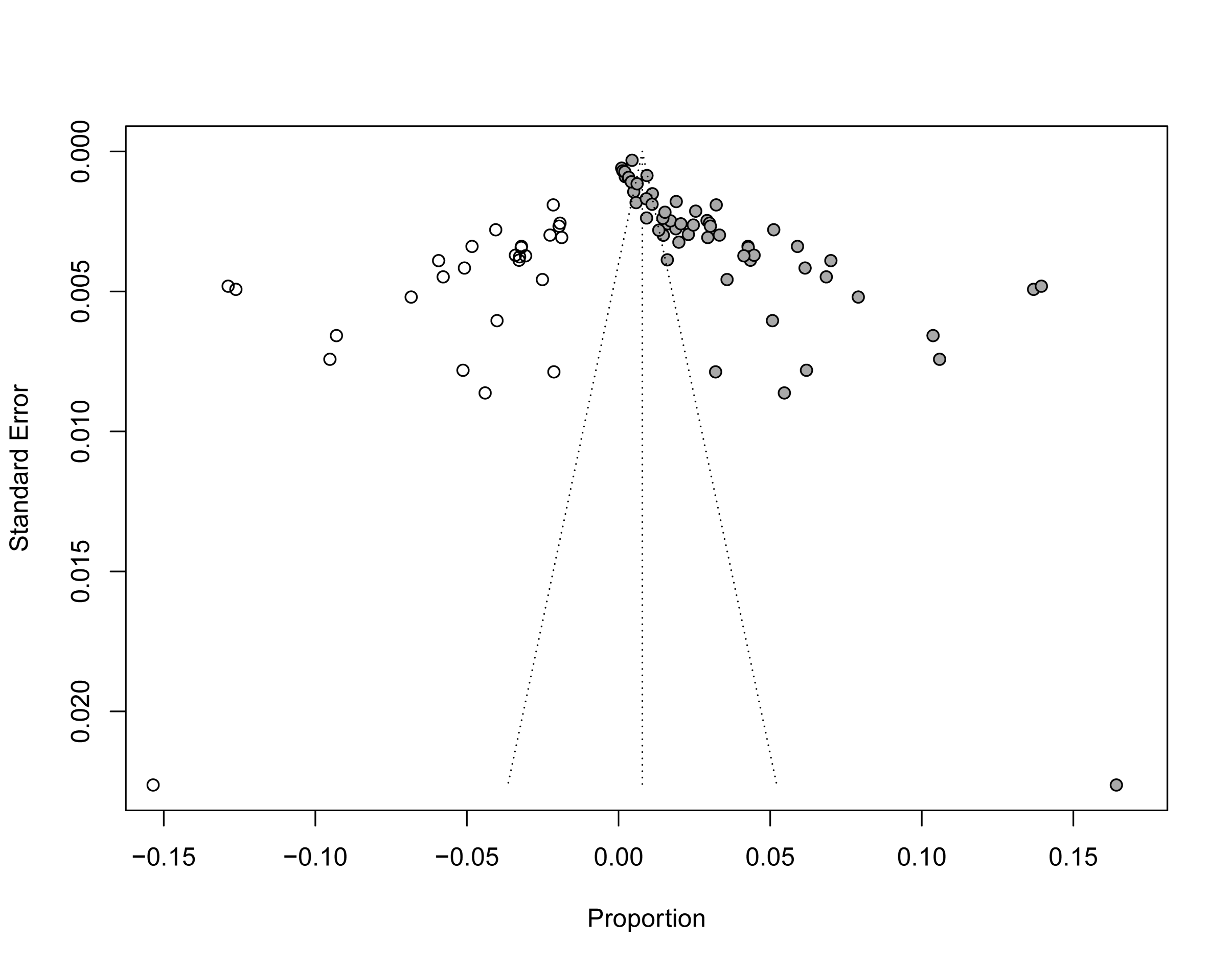

Supplement: Supplementary file 4 [file medi-102-e35644-s004.tif]

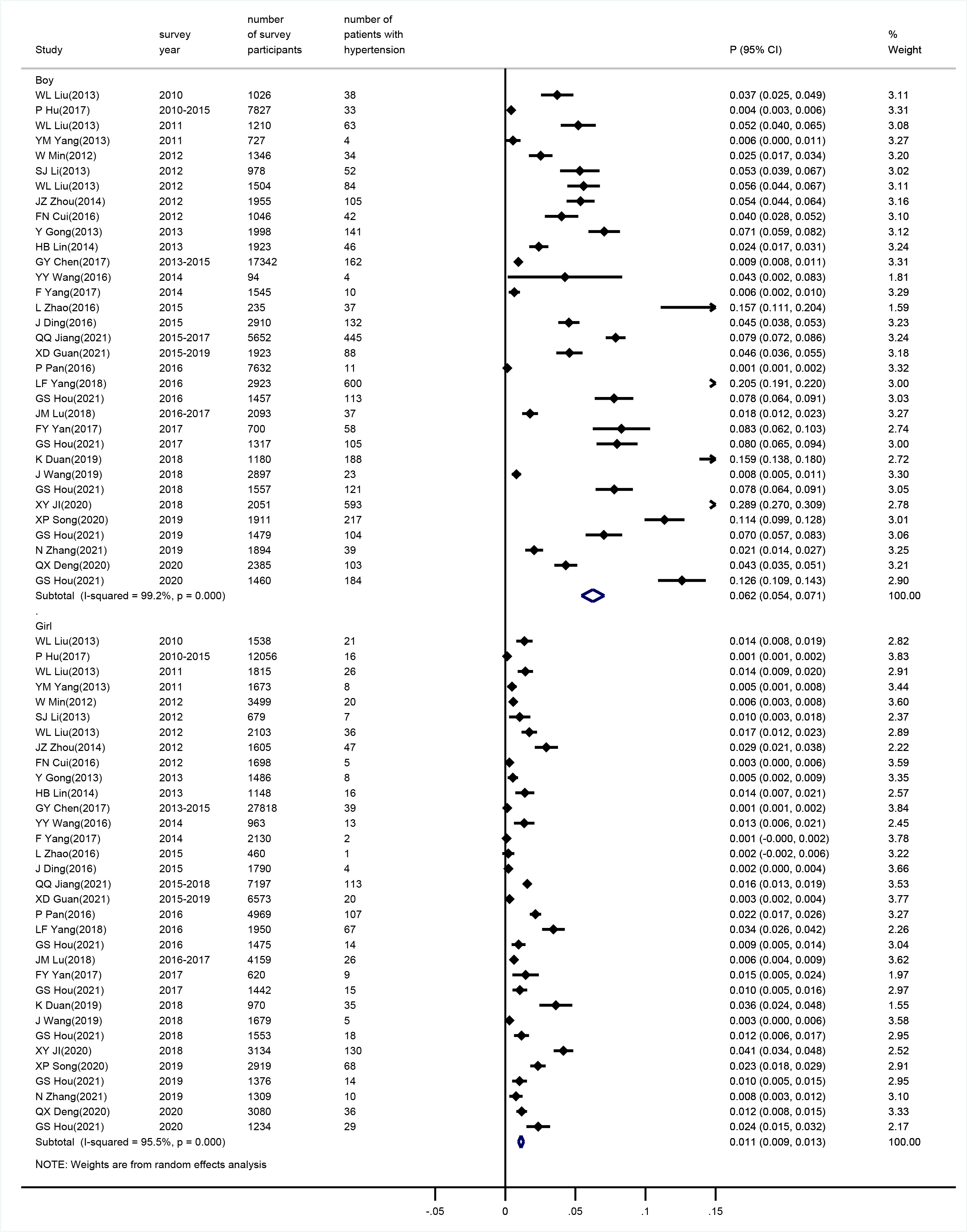

Supplement: Supplementary file 5 [file medi-102-e35644-s005.tif]

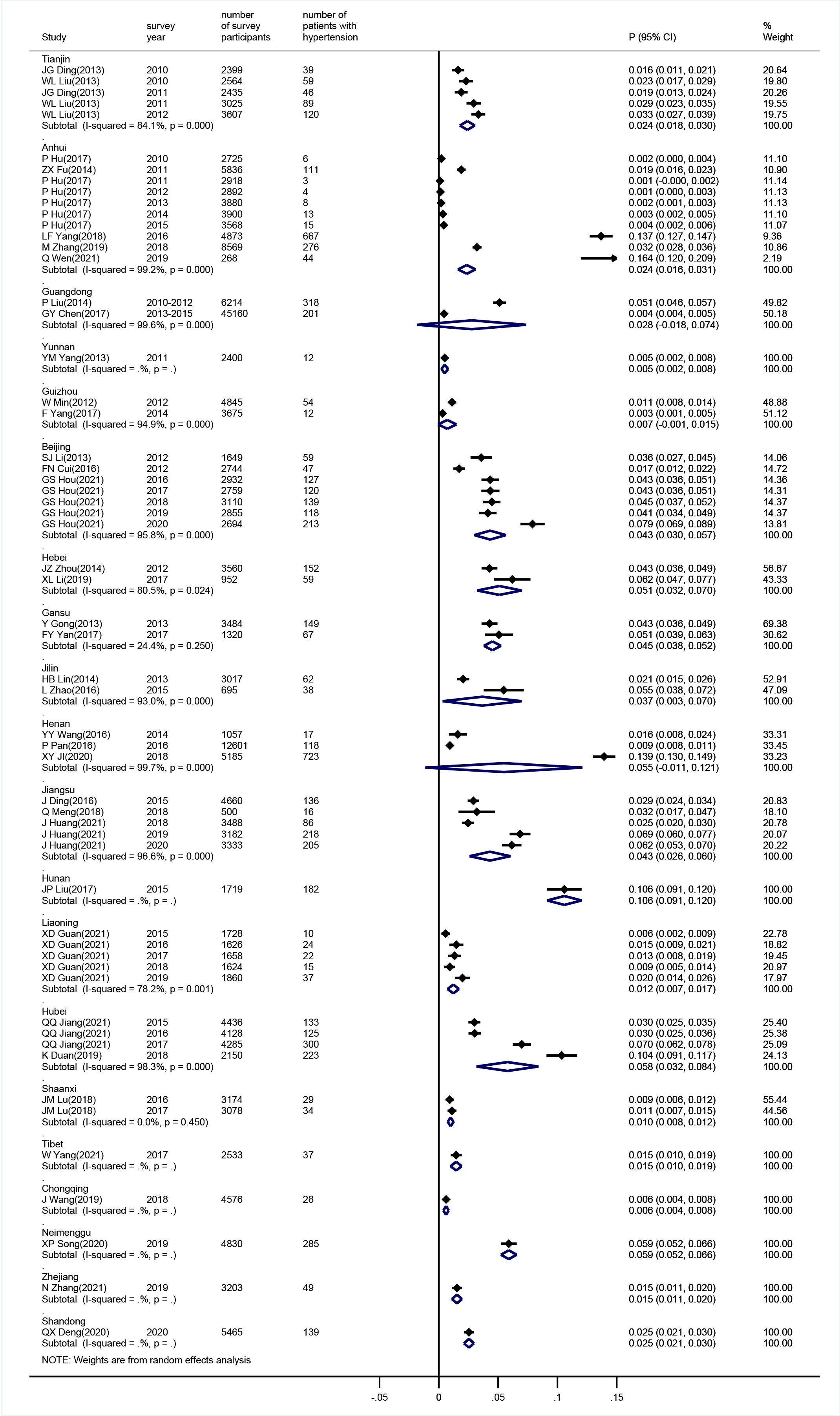

Supplement: Supplementary file 6 [file medi-102-e35644-s006.tif]

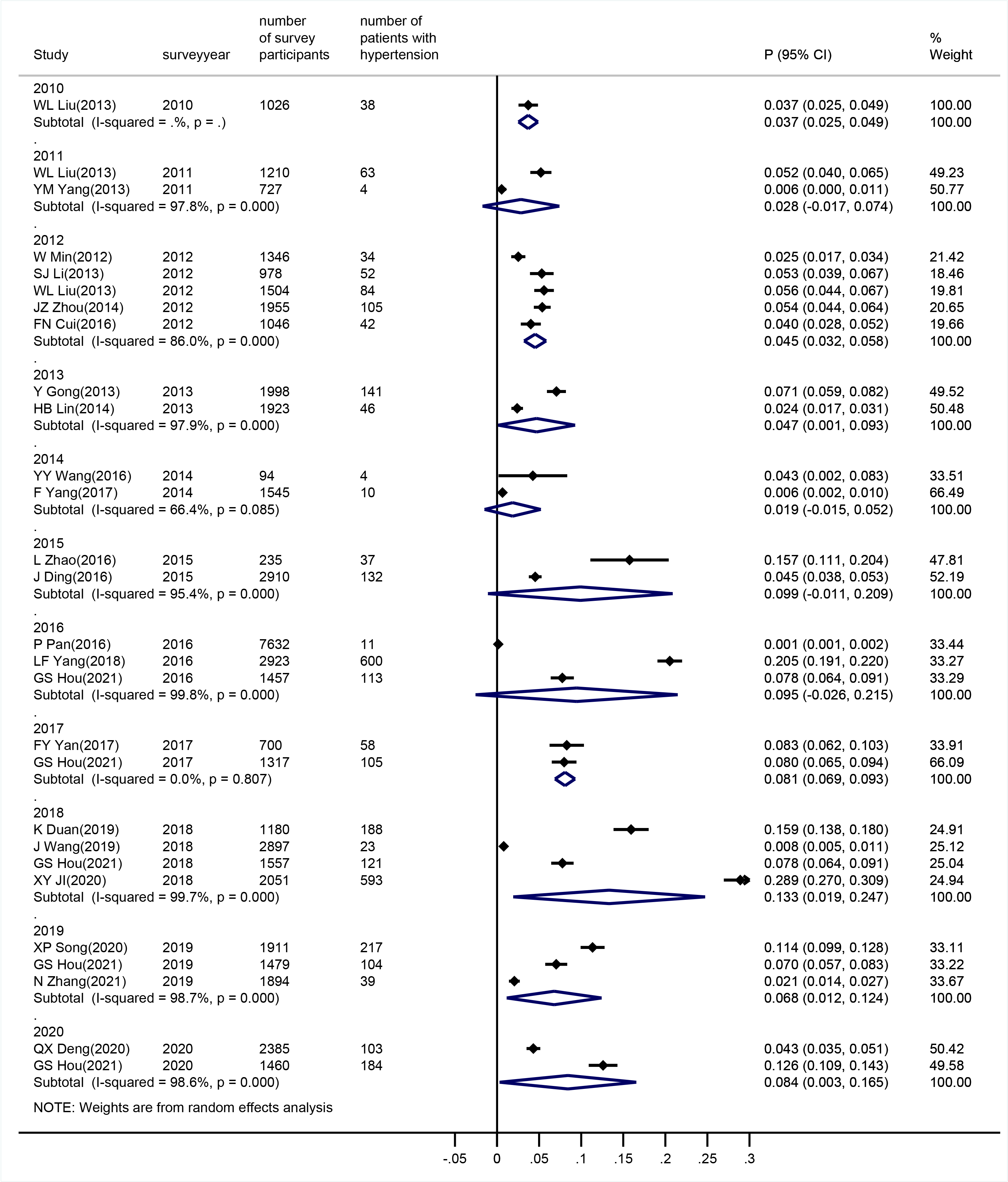

Supplement: Supplementary file 7 [file medi-102-e35644-s007.tif]

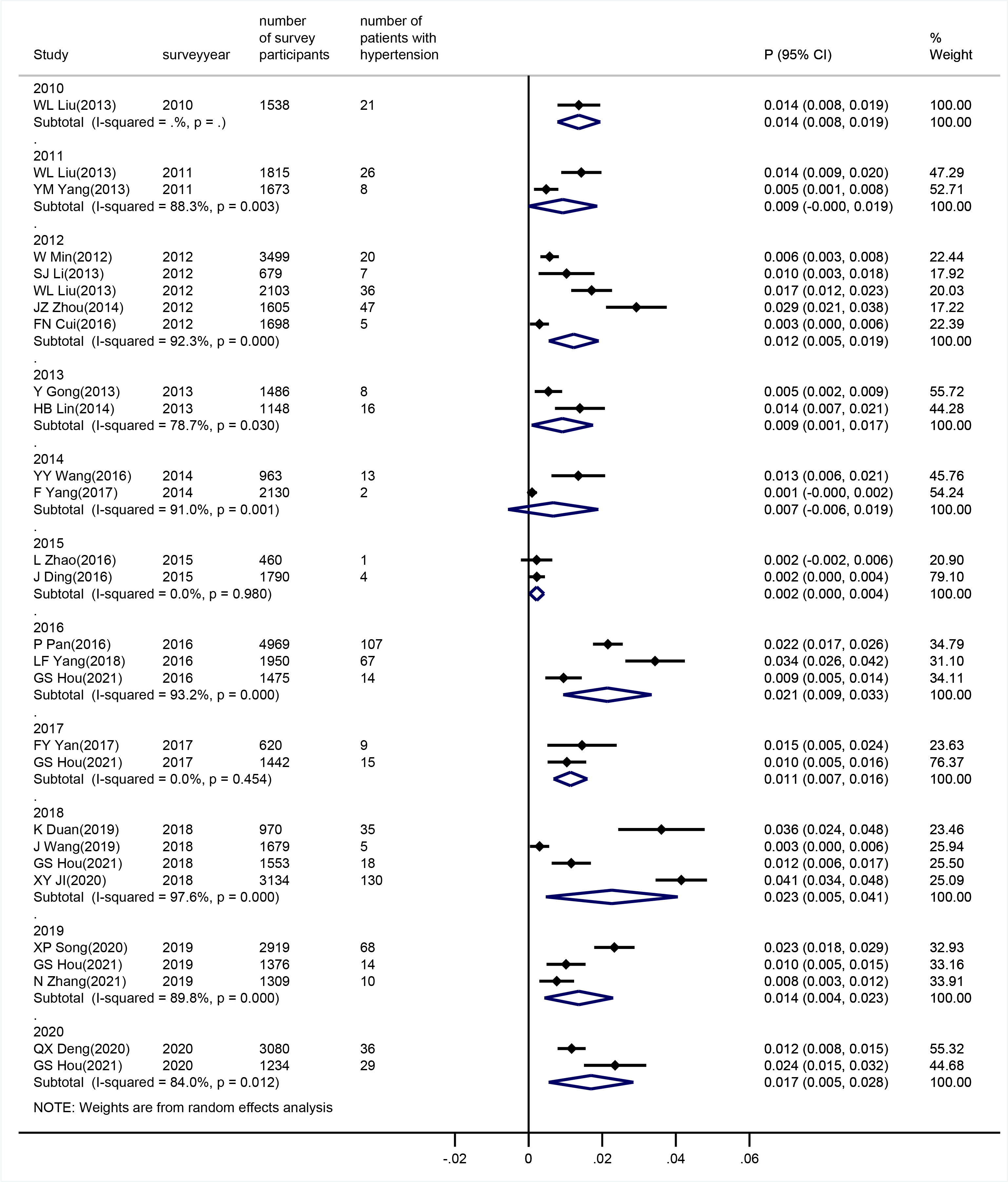

Supplement: Supplementary file 8 [file medi-102-e35644-s008.tif]

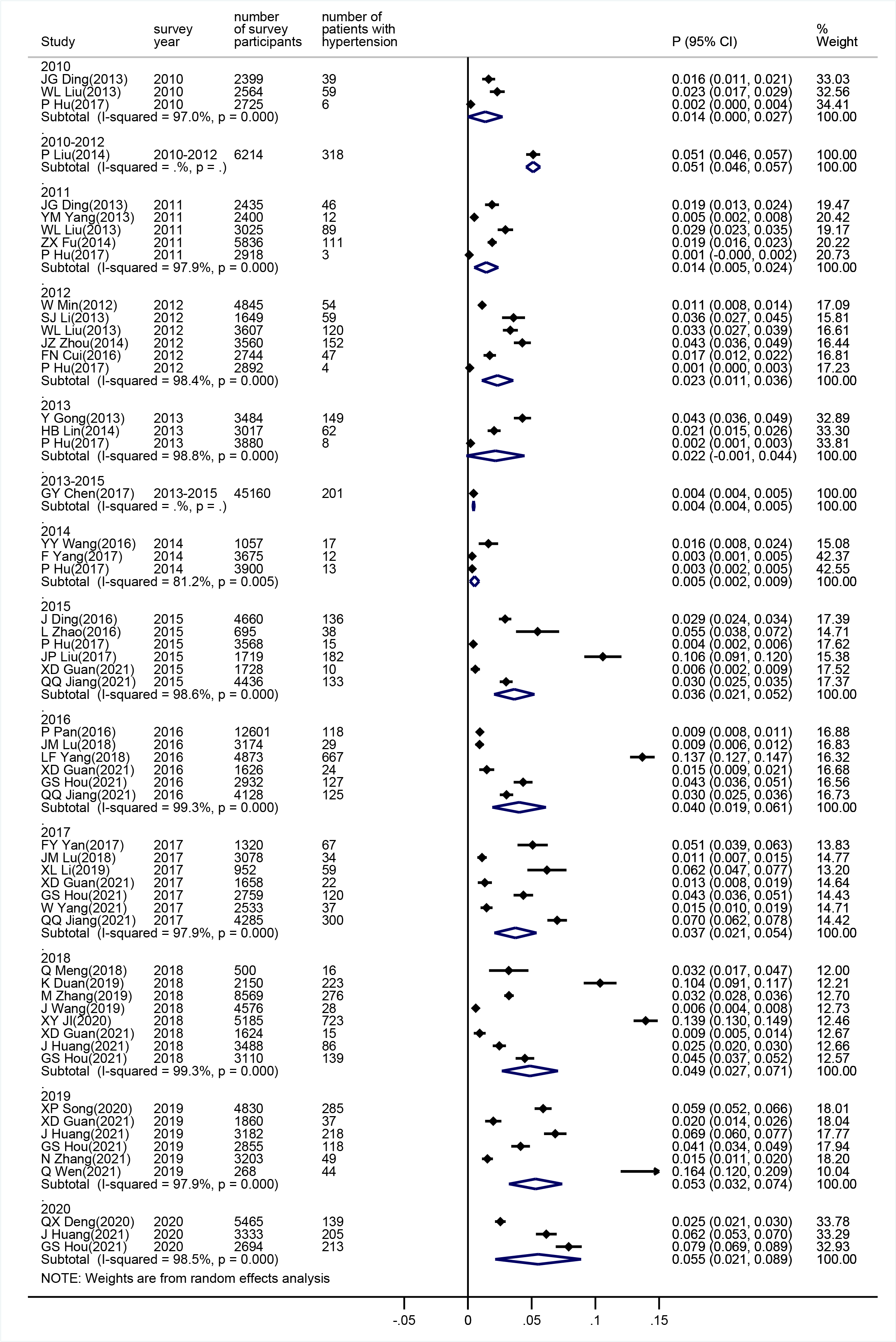

Supplement: Supplementary file 9 [file medi-102-e35644-s009.tif]
